# Surface plasmon resonance unveils important pitfalls of enzyme-linked immunoassay for the detection of anti-infliximab antibodies in patients’ sera

**DOI:** 10.1038/s41598-021-94431-x

**Published:** 2021-07-22

**Authors:** Marten Beeg, Cesare Burti, Eleonora Allocati, Clorinda Ciafardini, Rita Banzi, Alessandro Nobili, Flavio Caprioli, Silvio Garattini, Marco Gobbi

**Affiliations:** 1 Istituto di Ricerche Farmacologiche Mario Negri IRCCS, Milano, Italy; 2 Gastroenterology and Endoscopy Unit, Ospedale Papa Giovanni XXIII, Bergamo, Italy; 3 Unità Operativa di Gastroenterologia ed Endoscopia, Fondazione IRCCS Ca’ Granda Ospedale Maggiore di Milano Policlinico, Milano, Italy; 4 Department of Pathophysiology and Transplantation, Università Degli Studi di Milano, Milano, Italy; 5 Laboratory of Pharmacodynamics and Pharmacokinetics, Istituto di Ricerche Farmacologiche Mario Negri IRCCS, Via Mario Negri 2, 20156 Milano, Italy

**Keywords:** Inflammatory bowel disease, Laboratory techniques and procedures, Biological therapy, Surface plasmon resonance

## Abstract

Measurements of serum concentrations of therapeutic antibodies and anti-drug antibodies (ADA) can support clinical decisions for the management of non-responders, optimizing the therapy. In the present study we compared the results obtained by classical ELISA and a recently proposed surface plasmon resonance (SPR)-based immunoassay, in 76 patients receiving infliximab for inflammatory bowel diseases. The two methods indicated very similar serum concentrations of the drug, but there were striking differences as regards ADA. All the sera showing ADA by ELISA (14) also showed ADA by SPR, but the absolute amounts were different, being 7–490 times higher with SPR, with no correlation. Eight patients showed ADA only with SPR, and these ADA had significantly faster dissociation rate constants than those detectable by both SPR and ELISA. The underestimation, or the lack of detection, of ADA by ELISA is likely to reflect the long incubation steps which favor dissociation of the patient’s low-affinity ADA, while the commercial, high-affinity anti-infliximab antibodies used for the calibration curve do not dissociate. This problem is less important with SPR, which monitors binding in real time. The possibility offered by SPR to detect ADA in patients otherwise considered ADA-negative by ELISA could have important implications for clinicians.

## Introduction

Patients receiving therapies with monoclonal antibodies (mAb) often differ widely in their drug pharmacokinetics, and inadequate drug concentrations are a major cause of primary or secondary loss of response^1,2^. The latter may also be a consequence of the development of anti-drug antibodies (ADA) which can affect clinical efficacy by either neutralizing the therapeutic antibodies or increasing their clearance^3–5^. Thus, measurements of serum concentrations of the mAb and corresponding ADA (therapeutic drug and immunogenicity monitoring, TDIM) can support informed decisions for the management of non-responders, helping clinicians optimize dosage regimens or switching new therapeutic strategies, reducing unnecessary interventions^6,7^. Given the high cost of mAb, better use of these drugs would have a significant impact on health budgets.

The efficacy of TDIM for improving patients’ outcomes and reducing costs has been mainly investigated in patients with inflammatory bowel diseases, treated with the anti-TNFα monoclonal antibody infliximab (IFX)^8–10^. Many studies showed positive correlations between IFX concentrations and the outcomes of therapy^11–14^ and the incidence of immunogenicity on long-term drug efficacy^15–19^. Clinical- and cost-effectiveness aspects of algorithms based on the knowledge of drug and ADA levels, in comparison with the trial and error approach, have also been claimed, according to randomised clinical trials^20–23^. Thus, guidelines recommend TDIM as a reactive strategy when patients develop a loss of response^24–26^, although it has not yet been commonly adopted in routine practice.

Different bioanalytical assays are being used for TDIM, including enzyme-linked immunosorbent assays (ELISA)^11,17,18,27,28^, radioimmunoassays^29^, electrochemiluminescent immunoassays^30^, reporter gene assay^31^, homogeneous mobility shift assays^32^, with ELISA being the most popular. The variety of methods and thresholds applied^7,10,31^ and the limited or contradictory^33^ evidence of the superiority of TDIM over empiric decisions call for further research^34^. We recently characterized and validated an analytical assay to measure serum concentrations of IFX and the corresponding ADA, based on surface plasmon resonance (SPR)^35^. SPR is widely used to study *in real time* the interaction between two *unlabeled* molecules, one immobilized on a sensor chip, and the other flowing through a microfluidic system over the chip surface^36^. In this SPR assay the patient’s serum flows over parallel surfaces of the *same* sensor chip coated with TNFα and IFX, allowing specific binding of the serum IFX and ADA, respectively. This binding results in *immediate* and concentration-dependent SPR signals, from which IFX and ADA concentrations are determined *simultaneously* on calibration curves. Thus, in comparison to ELISA and the other techniques proposed so far, SPR has the obvious advantages that it does not require labeled compounds and that it avoids long incubation/separation/detection steps, reducing complexity and the related variability. We demonstrated these advantages of SPR through rigorous characterization and validation of the assay performances^35^.

Analysis of the serum of 15 patients treated for inflammatory bowel diseases (IBD), showed that the trough IFX levels measured by SPR were well superimposable with those given by a commercial ELISA^35^. However, there were striking differences as regards ADA. SPR indicated absolute ADA concentrations much higher—by one or two orders of magnitude—than those indicated by ELISA, with no correlations between the results of the two methods. However, SPR detected ADA in all but one patient’s sera where ELISA detected ADA, even when the levels with ELISA should have been too low for SPR detection. We suggested that the patients’ ADA levels were underestimated by ELISA because they have a faster dissociation rate constant (and thus lower affinity for IFX) than the ADA used for the calibration curve. Thus, SPR data indicated potential pitfalls of ELISA, i.e. that patient’s ADA may significantly dissociate from IFX during this ELISA incubation step while the ADA used for the calibration does not.

This hypothesis is consistent with previous data showing that the limit of detection of ELISA is inversely proportional to the affinity of the tested ADA^37^ and ELISA may fail to detect low-affinity antibodies^38^. Since SPR measures the binding events in a much shorter time than ELISA, its results can be expected to be much less affected. In line with this, Beeg et al.^35^ showed that SPR detected ADA in a serum which seemed ADA-negative with ELISA, and these ADA had the fastest dissociation rate from IFX. This was the only ADA-positive serum in which IFX was also detected.

The possibility that ELISA, i.e. the most common technique used in clinical practice for TDIM, could miss the presence of ADA in some patients might have important consequences for correct interpretation of the clinical outcome, and/or for appropriate clinical decisions. Here we further investigated this possibility by comparing the results of ELISA and SPR in a much larger number of patients receiving IFX, and exploited the potential of SPR to clarify the kinetic reasons for the different detection of ADA with the two methods.

## Results

We analyzed the serum samples from 76 patients in maintenance therapy with IFX for IBD, either Crohn’s disease or ulcerative colitis (see Table S1 for their main characteristics). IFX trough levels and ADA serum concentrations were measured with a commercial ELISA (Theradiag’s LISA-TRACKER Duo Infliximab) and by SPR. The concentrations of IFX and ADA in each serum sample were determined by SPR in triplicate, with ex-novo preparation of samples and calibration curves, by two separate researchers with different experience, and the results confirmed that SPR is highly reproducible and robust (Suppl Fig. S1).

### IFX concentrations

**Figure 1:**
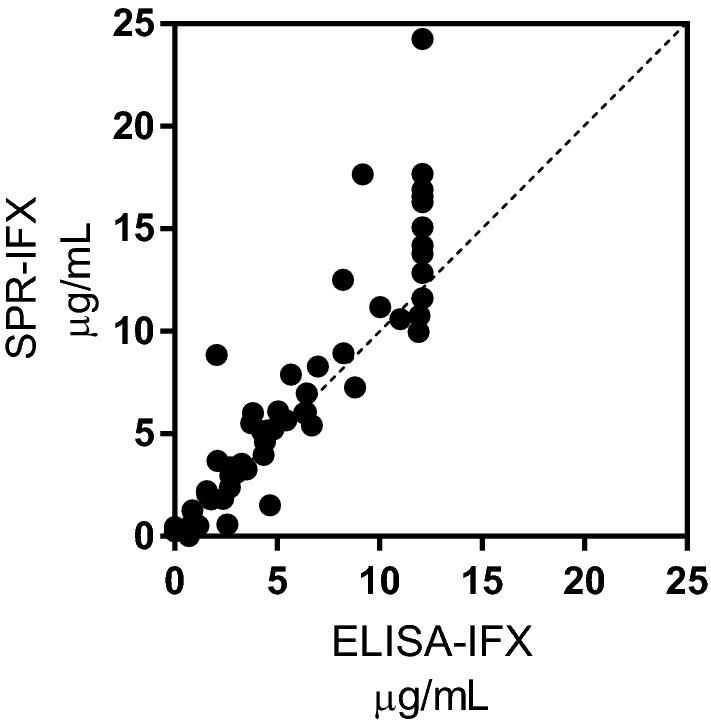
Concordance between serum concentrations of IFX determined by SPR and ELISA. The graph reports the values in 58 patients, i.e. those in which IFX levels were measurable by at least one method. The dotted line indicate the identity line. Bland–Altman analysis, carried out using GraphPad Prism version 7.00 for Windows (GraphPad Software, La Jolla California USA) estimated an “Average Bias” value, i.e. the average of the differences (computed after removal of those out-of-scale for ELISA) of 0.46 ± 1.93 (SD of the Bias), not significantly different from 0.

IFX was detectable in the sera of 57 and 56 patients by SPR and ELISA, respectively. The values with the two methods showed a very good concordance (Fig. 1).

**Figure 2:**
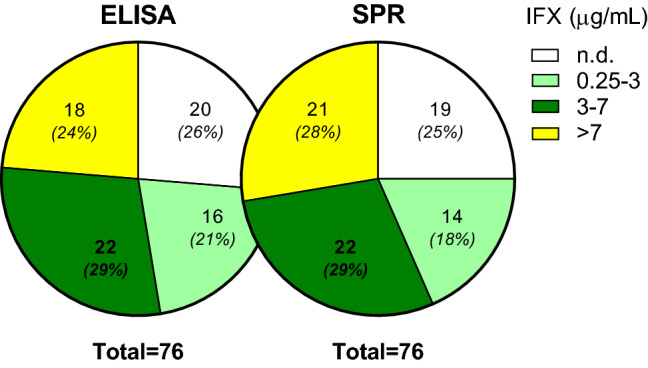
Number of patients grouped on the basis of IFX levels, detected by ELISA or SPR. N.d. indicates levels below the limit of detection (0.25 μg mL^−1^), and 3–7 (μg mL^−1^) indicates the therapeutic range.

Figure 2 shows the numbers of patients with IFX serum levels within the assumed therapeutic range (3–7 μg mL^−1^)^21^, and the numbers of with too low or too high levels, as identified with the two methods.

The results confirmed the very good correspondence between the two methods, although they were not exactly superimposable (Suppl Fig. S2). However, only two patients among those with IFX detectable by both methods had differences that induce the clinician to modify therapy: one patient (#67) had in-range values by ELISA (2.05 μg mL^−1^) but high values by SPR (8.85 μg mL^−1^); and one patient (#70) had too low SPR values (1.52 μg mL^−1^) and in-range ELISA values (4.65 μg mL^−1^).

In the whole set of data only 22 patients (29%) had IFX in the therapeutic range, independently of the analytical method. Similar proportions of patients (24–28%, depending on the method) had IFX levels exceeding the therapeutic range while quite a high proportion (43–47%) had too low values. About 25% of patients had undetectable IFX by both methods.

### ADA concentrations

ADA are expressed as μg Equivalents mL^−1^, to show that the ADA used for the calibration curves differ from those produced by the patients.

**Figure 3:**
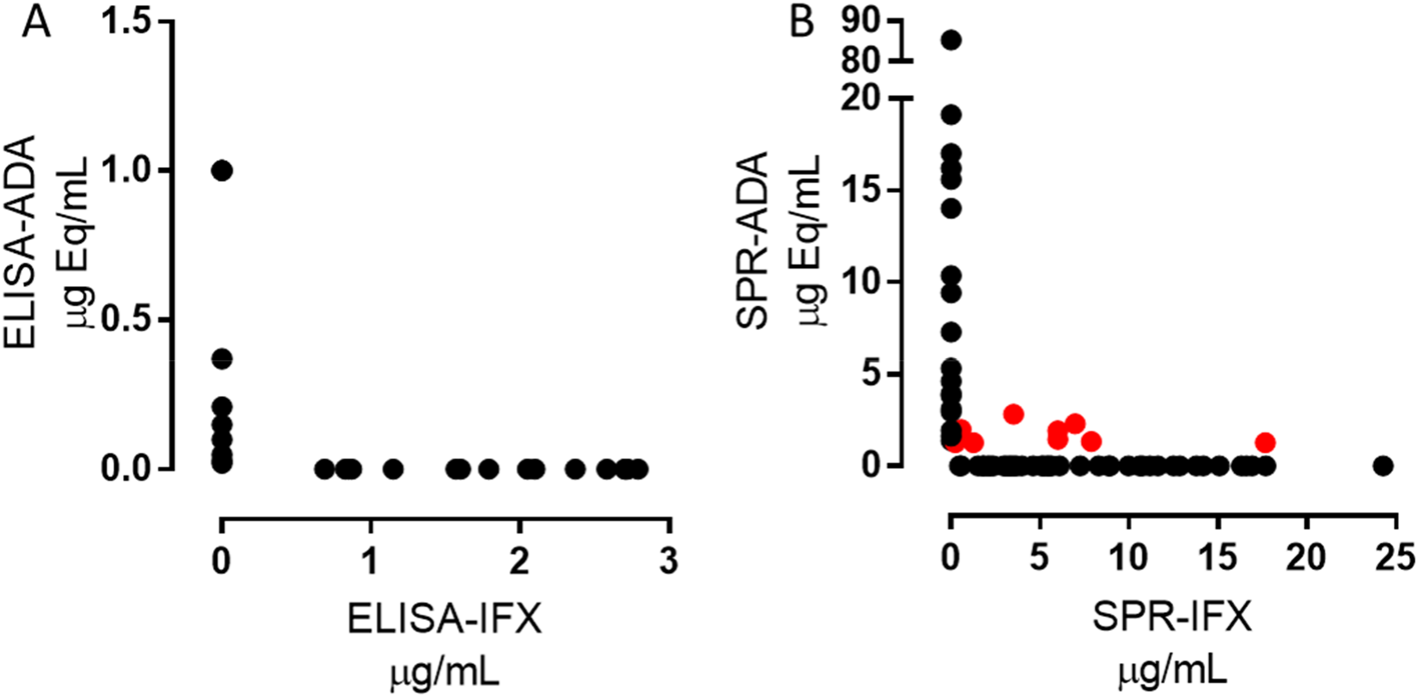
Levels of anti-IFX antibodies (ADA) and IFX, measured by: (**A**) ELISA in the plasma of 36 patients (i.e. those with IFX < 3 μg mL^−1^) and (**B**) SPR (76 patients). Red dots highlight the patients in which ADA were detectable despite measurable IFX levels. ADA are expressed as μg Equivalents mL^−1^, to indicate that the ADA used for the calibration curves are different from those produced by the patients.

The ELISA we used is a drug-sensitive assay that detected ADA only in serum from 36 patients with low IFX (< 3 μg mL^−1^). ADA were detectable in 14 of these (18% of total, Fig. 3A), all with undetectable IFX; in contrast, no ADA were found in the 16 patients with detectable IFX. Six patients had no IFX or ADA.

All 76 patients’ sera were analyzed for ADA by SPR, previously described to be drug-tolerant^35^. ADA were detectable in 28 (37%) (Fig. 3B). All the patients with undetectable IFX (19) had ADA, whose levels varied widely (1.4–85 μg Eq mL^−1^). Strikingly, however, ADA were also clearly detected in 9 patients with detectable IFX, six of them with IFX > 3 μg mL^−1^ (red in Fig. 3B). These data confirm that ADA detection by SPR is “drug-tolerant”. In fact, we previously showed that in our SPR assay, which includes acidic pre-treatment, 8 μg IFX mL^−1^ undiluted serum had no interfering effect on the determination of 5 μg ADA mL^−1^ undiluted serum^35^. This is a well-known limitation of ELISA that prevents the measurement of ADA in the presence of IFX.

On the other hand, the detection of IFX binding to immobilized TNFα in ADA-positive samples could be due to either too-low ADA concentration, or the presence of not-neutralizing ADA. Thus, we examined the neutralizing properties of the ADA detected by SPR. All the ADA-positive serum samples were spiked with 8 µg mL^−1^ IFX, and the SPR binding signal to immobilized TNFα was compared to the SPR binding signal observed with 8 µg mL^−1^ IFX in the absence of ADA. Thus, neutralizing antibodies will lower the IFX-dependent binding signal whereas non-neutralizing ones will not. The data suggest that most of the ADA detected in the IFX-negative samples were neutralizing, as expected, whereas the ADA in IFX-positive samples (9 patients, red in Fig. 3B) appeared to be not-neutralizing.

All the patients’ sera showing ADA with ELISA (n = 14) also showed ADA with SPR. However, as previously reported and discussed^35^, the absolute amounts of ADA clearly differed, being 7–490 times higher with SPR, and no correlation was found between the levels measured with the two methods.

We identified 8 patients’ sera ADA-positive by SPR and ADA-negative by ELISA. To clarify this difference, we looked more closely at the sensorgrams obtained when injecting the serum samples containing the different patients’ ADA over immobilized IFX. SPR can follow the association and dissociation phases in real time, and this is a further value of this method. While the association rate constant (kon) cannot be determined because of lack of information on the real concentration of the ADA (we can only estimate an “equivalent” concentration, determined on the calibrator ADA), the dissociation rate constant (koff), expressed in s^−1^, can be measured by fitting the sensorgram in the dissociation phase.

**Figure 4:**
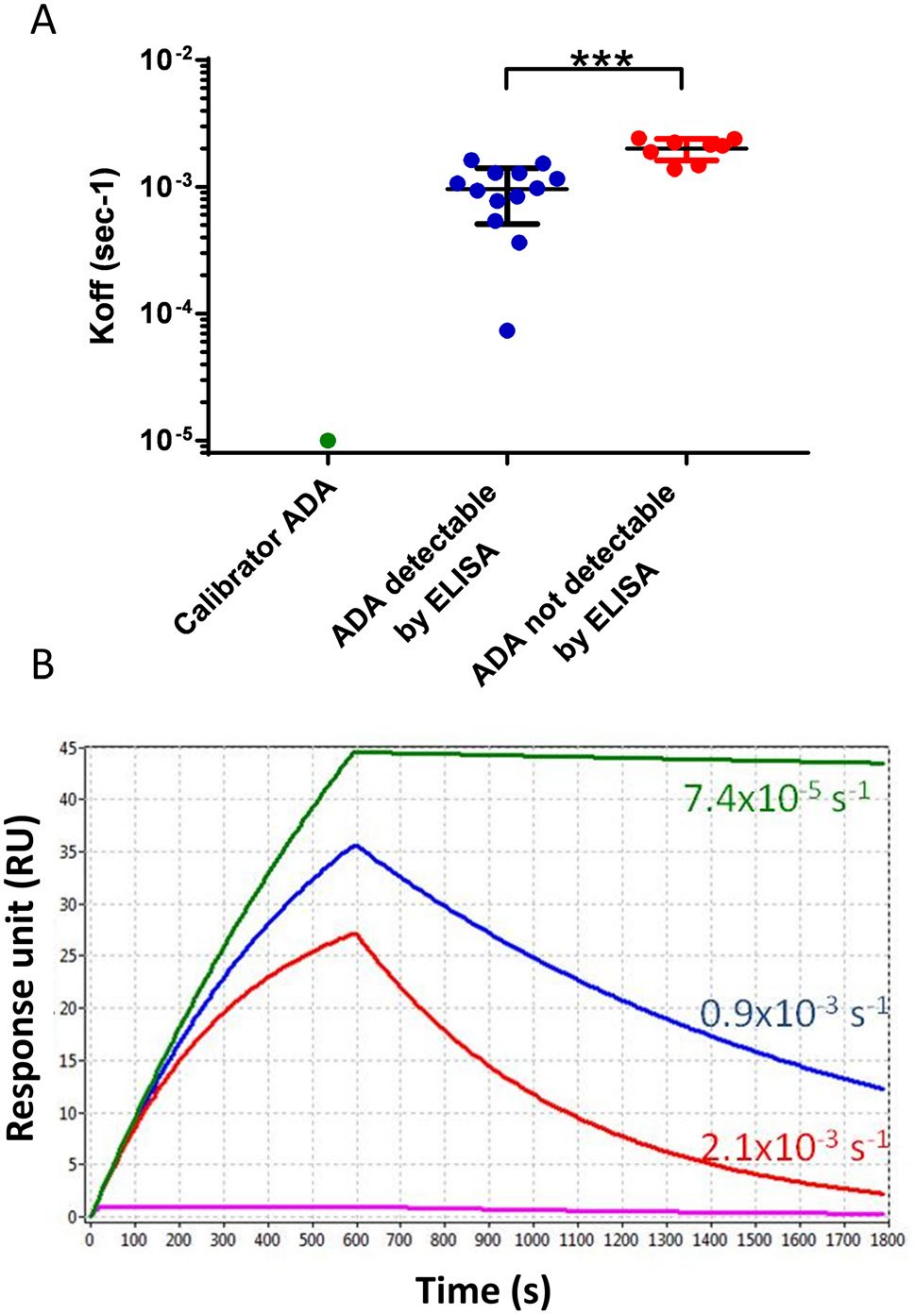
Upper panel shows the dissociation rate constants (koff, in s^−1^) determined by SPR for the patients’ ADA; each point represents a single patient. Only some of these patients’ ADA were detectable by ELISA (blue), and these had significantly slower koff than the ADA not detectable by ELISA (red) (p < 0.001 Student’s T test). The koff value of the commercial anti-IFX antibody used for the calibration curve is shown for comparison (green). Lower panel shows the sensorgrams simulating the SPR binding signals of three different ADA, with identical concentration (1 × 10^–8^ M) and kon (1 × 10^5^ M^−1^ s^−1^) but different koff, corresponding to the mean values shown in the upper panel.

The koff of the patients’ ADA varied widely, from 3.0 × 10^–3^ s^−1^ (i.e. 0.3% of the bound ADA dissociate per second) to 7.4 × 10^–5^ s^−1^ (Fig. 4). More importantly, the patients’ ADAs detectable only by SPR, but not ELISA, had a significantly (p < 0.001) faster dissociation rate constant (2.1 × 10^–3^ s^−1^, 95% CI 1.7–2.3 × 10^–3^) than the ADAs detectable by both SPR and ELISA (0.9 × 10^–3^ s^−1^, 95% CI 0.7–1.2 × 10^–3^) (Fig. 4A). Figure 4B shows simulated sensorgrams as a visual and practical representation of the impact of the detected koff on the dissociation phase. Within a time-frame of 20 min, the ADA with a koff of 2.1 × 10^–3^ s^−1^ (the mean koff of the ADAs detectable by SPR but not ELISA) almost completely dissociated from immobilized IFX, whereas those with a koff of 0.9 × 10^–3^ s^−1^ dissociated only 65%.

**Figure 5:**
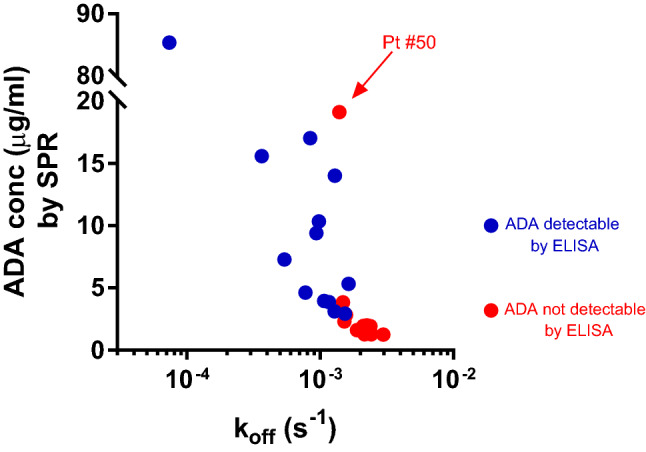
Relation between the koff of the patients’ ADA, determined by SPR, and the SPR binding signals (transformed into μg Eq mL^−1^ according to the calibration curve).

The sensorgrams in Fig. 4B were generated assuming identical kon (1 × 10^5^ M^−1^ s^−1^) and analyte concentration (1 × 10^–8^ M). These sensorgrams therefore illustrate how differences in koff also affect the association phase (except the very early part, when dissociation of the bound analyte is still negligible). In particular, the faster the dissociation rate, the lower the binding signal measured at the end of the association phase. These data may partly explain the apparent correlation between the koff values of the patients’ ADA and the corresponding SPR binding signals (transformed into μg Eq mL^−1^ in Fig. 5). However, the SPR binding signals are also dependent on the concentration and the kon of the patients’ ADA (for example, the high SPR binding signal with the fast dissociating ADAs of patient #50, suggest a high concentration or a very fast kon).

## Discussion

The present study, analyzing the sera of 76 patients under maintenance treatment with IFX for IBD, confirms and extends our previous data in 15 patients^35^, and provides new information with potential clinical relevance.

In particular:

(i) the reproducibility and the reliability of the SPR assay for TDIM is confirmed. SPR allows the simultaneous measurement of IFX and the corresponding ADA within one injection cycle; dozens of consecutive injections can be carried out on the same chip thanks to the highly efficient procedure for surface regeneration; and a cycle of injection of serum samples and chip regeneration takes approximately 20 min. These all allow a robust, rapid, drug-tolerant^35^ assay, with costs competitive with those of ELISA.
(ii) The very good correspondence between the serum IFX concentrations measured with SPR and those measured by ELISA is replicated. These data confirm that when calibration curves are built with the same analyte to be measured (i.e. IFX), SPR gives the same results as ELISA. The IFX serum levels showed wide inter-individual variability in the patients tested, with values from 0 to 25 μg mL^−1^. IFX levels were within the therapeutic range only in about a quarter of patients, while they were exceedingly high in another quarter and too low in half; about 25% of patients showed undetectable IFX by both methods. These data confirm previous data^11,15^ and suggest the usefulness of further exploration of TDIM based on different approaches.
(iii) Fourteen patients were ADA-positive with both ELISA and SPR. However, the ADA concentrations were strikingly different, − 7–490 higher than with SPR − confirming previous data^35^. Our hypothesis is that ELISA may markedly underestimate ADA concentrations due to the different affinity between patients’ ADA and the commercial anti-IFX antibody used as calibrator^35,37^. In fact, SPR data showed that the latter has a dissociation rate constant of < 1 × 10^–5^ s^−1^ (i.e. pseudo-irreversible binding to immobilized IFX) whereas all the patients’s ADA had much faster dissociation rate constants, rfrom 3.0 × 10^–3^ to 7.4 × 10^–5^ s^−1^. Thus, in the ELISA, the calibrator will not dissociate during the long incubation with the secondary antibody, after the unbound ADA have been washed away, whereas faster-dissociating ADA may dissociate (see simulations in Fig. 5). It follows then, that a low concentration of the calibrator will produce the same ELISA signal as a high concentration of faster-dissociating patients’s ADA, resulting in significant underestimation of the concentration of the latter. The difference between ELISA and SPR results, which indicate much higher concentrations of patients’ ADA, suggest that SPR is less affected. This is likely because SPR measures the binding events in real time and in a very short time, avoiding the long incubation steps of ELISA. So SPR data unmask a major drawback of ELISA: the extent of the ELISA underestimation depends on ADA’s binding properties, and thus cannot be predicted.
(iv) Besides the 14 patients whose ADA where detectable by both SPR and ELISA (although underestimated with the latter), we also found 8 patients who had ADA with SPR only, not ELISA. Thus SPR detected ADA in a larger proportion of patients than ELISA, considering either the total proportions of patients (36.8% vs 18.4%, p = 0.01), or the patients with IFX concentrations < 3 μg mL^−1^ (61.1% vs 38.8%, p = 0.059), the latter being the ideal condition for ELISA. According to a recent survey of 80 studies, the proportion of ADA-positive patients varies widely, from 4.8 to 79% depending on the assay^39^. These data support the importance of using a well-characterized and reliable test for ADA.
(v) The ADA only detectable by SPR had significantly faster dissociation rate constants than those detectable also by ELISA. This further supports the importance of ADA’s koff: only the slower-dissociating ADAs can be detected (although underestimated) by ELISA while the faster-dissociating ones cannot be detected at all, but are present in patients’ sera.
(vi) The ADA levels given by SPR in the sera where ELISA did not detect ADA were generally low, though clearly measurable (Fig. 3B, red dots, and Fig. 4). This could be a direct consequence of the fast dissociation rate constant of these ADA, which also slows the association phase (see simulation in Fig. 5). Furthermore, the association phase depends on both the concentration and the association rate constant (kon, M^−1^ s^−1^) of the ADA, which are both unknown and cannot be distinguished by sensorgram analysis. Thus, a similar SPR signal at the end of the association phase (i.e. a similar apparent concentration estimated from the calibration curve) might be due to − for example − concentration 10 and kon 1, or concentration 1 and kon 10. This highlights the important point that the concentrations taken from the calibration curve with a commercial ADA having different binding constants, might be considered as only apparent, being affected by the binding constants of the patients’ ADA, which are unknown. This is true for SPR but also for any other analytical method employing a calibrator different from the analyte, including ELISA. Determining the exact concentration of the patient’s ADA would require their purification and their use as calibrators.

Nevertheless, for the reasons set out in points (iii) and (iv), SPR appeared superior to ELISA in that it recognized ADA in sera identified as ADA-negative by ELISA.

(vii) Although limited by the explorative nature of our study, the possibility offered by SPR to detect ADA in patients otherwise considered ADA-negative by ELISA could have important implications for clinicians. This might be the case for three patients with active disease (# 49, 50 and 62) who showed no IFX by either methods: according to ELISA results (no ADA present) one could envisage the need to increase the IFX dose, but this would be deleterious if ADA are actually present (as for the SPR result); notably, one of them (#50) had a very high ADA-dependent SPR signal. Some other patients (# 56, 15, 52, 13) were in remission despite low or undetectable levels of IFX, and in these cases too ELISA did not detect ADA whereas SPR did. These patients may benefit from stopping treatment because presumably their clinical remission is not linked to the drug, and SPR results could support this decision so as to avoid the potential side effects associated with ADA. Finally, SPR but not ELISA detected ADA in the serum of five patients (# 8, 37, 73, 79, 80) with more than adequate IFX levels (≥ 6 µg mL^−1^) and no disease activity: in this case the information provided by SPR could suggest adding an immunomodulator to prevent ADA adverse effects.

In summary, even though SPR and ELISA give comparable IFX levels, ELISA fails to detect ADA in some patients, in particular the ADA with faster dissociation rate constants. This can possibly lead to incorrect evaluation of the patient’s situation and/or suggesting wrong therapeutic interventions. Since the specific features of SPR can overcome these limitations, SPR-based assays should be considered a reliable alternative to ELISA.

## Methods

### Patients

We analyzed the serum samples from 76 patients in maintenance therapy with IFX (Remsima®, Celltrion; Inflectra®, Pfizer) for IBD, either Crohn’s disease or ulcerative colitis, at the Fondazione IRCCS Cà Granda Ospedale Maggiore Policlinico (Milan, Italy) between April 2018 and July 2019. Inclusion criteria were adult age and the beginning of IFX therapy at least 8 weeks before serum sampling.

The study was approved by the Ethical Committee of the Fondazione IRCCS “Cà Granda” (n. 1310/2019). All patients provided informed consent. All procedures were in accordance with the ethical standards of the institutional and/or national research committee and with the 1964 Helsinki declaration and its later amendments, or comparable ethical standards.

Baseline characteristics of the patients are summarized in Table S1. Medical information about patients were retrospectively extracted from medical records. Because of the study’s retrospective nature and the lack of routine clinical score recording, clinical activity was based on the judgment of the treating physicians, as documented in the patients’ charts.

Blood samples were taken just before the infusion of a maintenance dose, to obtain drug trough levels, and sera were immediately obtained and stored at − 80° until analysis.

To assess biochemical and endoscopic activity we used respectively C-reactive protein (CRP) and colonoscopy reports, considering CRP obtained two months before or after the date of sampling for TDIM, and for endoscopic activity reports obtained six months before or after.

### ELISA

IFX and ADA were measured with CE-marked ELISA kits distributed by R-Biopharm AG (Germany), according to manufacturer’s guidelines. With RIDASCREEN®IFX, plasma IFX is captured by TNFα applied to the surface of the well and, after a washing step, detected by a highly specific anti-IFX monoclonal antibody (MA-IFX6B7) conjugated with horseradish peroxidase. For these analyses plasma samples were diluted 100 times. ADA were measured by RIDASCREEN® anti-IFX, with plasma samples diluted 200-fold. In this case, ADA were captured by IFX applied to the surface of the wells and, after a washing step, recognized by biotin-conjugated IFX which was eventually detectd by peroxidase-conjugated streptavidin. The manufacturer recommends measuring ADA when IFX concentrations in the serum sample are below 1 μg mL^−1^.

To expand the population and to investigate the assay’s performance also in patients with higher drug concentrations, ADA concentrations were measured in all serum samples with IFX below 3 μg mL^−1^.

### SPR

#### Control serum

Blood was taken from healthy volunteers and collected in *VACUETTE*® tubes with *Serum* Clot Activator (ref. 456,018, Greiner bio-one), then centrifuged. Serum was pooled, aliquoted and stored at − 80 °C.

#### Chemicals and reagents

The calibration curves of IFX and ADA were obtained with the IFX biosimilar CT-P13 (Hospira S.r.l., Naples, Italy) and the commercial anti-IFX antibody HCA-216 (Bio-Rad Laboratories, Segrate, Italy). The concentration of the stock solutions was checked by measuring the absorbance at 280 nm using an extinction coefficient of 217,440 M^−1^ cm^−1^^40^. 10 × Dulbecco-PBS was obtained from Euroclone S.p.A. (Pero, Italy). MgCl2, EDTA and Tween 20 were from Sigma-Aldrich (Milan, Italy). Water was provided in-house by a Milli-Q system (Millipore, Bedford, MA, USA).

#### SPR assay

The method described previously was used^35^. The SPR apparatus was the ProteOn XPR36 Protein Interaction Array system (Bio-Rad), which allows to immobilise different ligands on parallel strips of the same sensor surface. In this case, TNFα, IFX (Inflectra, as indicated), and IgG (control) were immobilized using amine-coupling chemistry on parallel strips of a GLC sensor chip (BioRad), according to manufacturer’s recommendation. After rotation of the fluidic system, analyte solutions were injected in parallel surfaces, so that they flowed on all the immobilized ligands, creating a multi-spot interaction array (see Fig. 1 in ref.^35^). Before injection, human sera containing IFX or ADA were, subjected to acidic pre-treatment. Firstly the samples were diluted 1:20 in 100 mM acetic acid pH 3 and incubated for 15 min at room temperature. Subsequently, the samples were diluted 1:1.5 in 0.5 M phosphate buffer pH 7.4, to a 30-fold overall sample dilution. The running buffer of the SPR instrument was 10 mM phosphate buffer containing 150 mM NaCl and 0.005% Tween 20 (PBST pH 7.4). Diluted patients’ sera or calibration standards flowed over immobilized ligands for three min at a rate of 30 µL min^−1^. Dissociation was measured in the following 7–11 min. All of these assays were done at 25 °C. The sensorgrams (time course of the SPR signal in RU) were normalized to a base-line value of 0. The signals observed in the surfaces immobilizing the ligands were corrected by subtracting the nonspecific response observed in the reference surface (“empty” surface for immobilized TNFα, and IgG for immobilized IFX). When indicated, the sensorgrams were fitted using the ProteOn analysis software to obtain the association and dissociation rate constants (kon and koff) and the equilibrium dissociation constant (K_D_).

The calibration curves included six-point calibrators in the range of 0.25–8 µg mL^−1^ control serum for IFX or 5–40 μg mL^−1^ control serum for the commercial anti-IFX antibody. Two separate runs with calibrators were carried out, one at the beginning and one at the end of each analytical session. Responses, expressed as the RU at the end of the dissociation phase, were plotted against the corresponding analyte concentration and the data were fitted using weighted (1/x^2^) linear regression. All calibration curves analyzed during method validation showed determination coefficients (r^2^) over 0.99; the accuracy of the back-calculated concentrations was always within the acceptance limits (± 15% of the nominal value).

ADA were expressed as μg Equivalents mL^−1^, to illustrate that the ADA used for the calibration curves are different from those produced by the patients.

## Supplementary Information

Supplementary Information.

## Data Availability

The datasets used and/or analyzed during the present study are available from the corresponding author upon reasonable request.

## Abbreviations

SPR: Surface plasmon resonance
ELISA: Enzyme-linked immunoassay
IFX: Infliximab
ADA: Anti-drug antibodies (anti-infliximab antibodies)
mAb: Monoclonal antibodies
CRP: C-reactive protein
TDIM: Therapeutic drug and immunogenicity monitoring
IBD: Inflammatory bowel diseases
