# Supplementary material for: Surface plasmon resonance unveils important pitfalls of enzyme-linked immunoassay for the detection of anti-infliximab antibodies in patients’ sera
Source: Sci Rep. 2021 Jul 22;11:14976. doi: 10.1038/s41598-021-94431-x (PMC8298394; doi:10.1038/s41598-021-94431-x)
Supplement: Supplementary file 1 — Supplementary Information. [file 41598_2021_94431_MOESM1_ESM.pdf]

SUPPLEMENTARY MATERIALS

Surface plasmon resonance unveils important pitfalls of enzyme-linked immunoassay for the detection of anti-infliximab antibodies in patients' sera

submitted to **Scientific Reports**

Marten Beeg[1], Cesare Burti[2], Eleonora Allocati[1], Clorinda Ciafardini[3], Rita Banzi[1], Alessandro Nobili[1], Flavio Caprioli[3], Silvio Garattini[1], Marco Gobbi[1*]

[*] Corresponding author:

Marco Gobbi
Laboratory of Pharmacodynamics and Pharmacokinetics
Istituto di Ricerche Farmacologiche Mario Negri IRCCS
Via Mario Negri 2
20156 Milano, Italy
marco.gobbi@marionegri.it

Table S1

Sex	
Male, no. (%)	50 (65,8%)
Female, no. (%)	26 (34,2%)
Mean age at diagnosis (yr, SD)	29. 1 ± 12.7
Mean duration of IFX therapy (mo, SD)	37.3 ± 30.1
IBD type, no. (%)	
Crohn's disease	53 (69.7%)
Ulcerative colitis	23 (30.3%)
Crohn's disease location, no. (%)	
ileum	12 (22.6%)
ileo-colon	31 (58.5%)
colon	10 (18.9%)
upper	3 (5.7%)
perianal disease	20 (37.7%)
Ulcerative colitis location, no. (%)	
Proctitis	
Left sided colitis	0 (0.0%)
Extensive	13 (56.5%)
	10 (43.5%)
Extraintestinal manifestations, no. (%)	7 (9.7%)
Concomitant IMM therapy, no. (%)	19 (25.0%)
IFX therapy regimen, no. (%)	
Standard	51 (67.1%)
Optimized	25 (32.9%)

Table S1. Characteristics of the 76 patients 1 IMM = immunomodulator (azathioprine, methotrexate). IFX standard regimen = 5mg/kg every 8 weeks, optimized regimen = 10 mg/kg and/or frequency shorter than 8 weeks.

Suppl. Fig 1

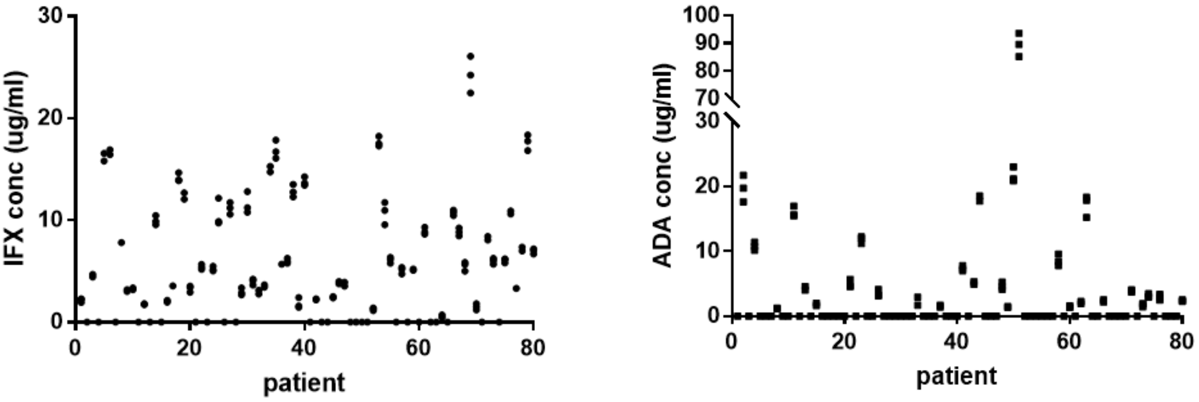

Suppl. Fig 1 *Inter-assay reproducibility of the SPR assay. The graphs show the results for each serum sample tested in triplicate, with ex-novo preparation of samples and calibration curves, by two separate researchers with different experience.*

Suppl. Fig 2

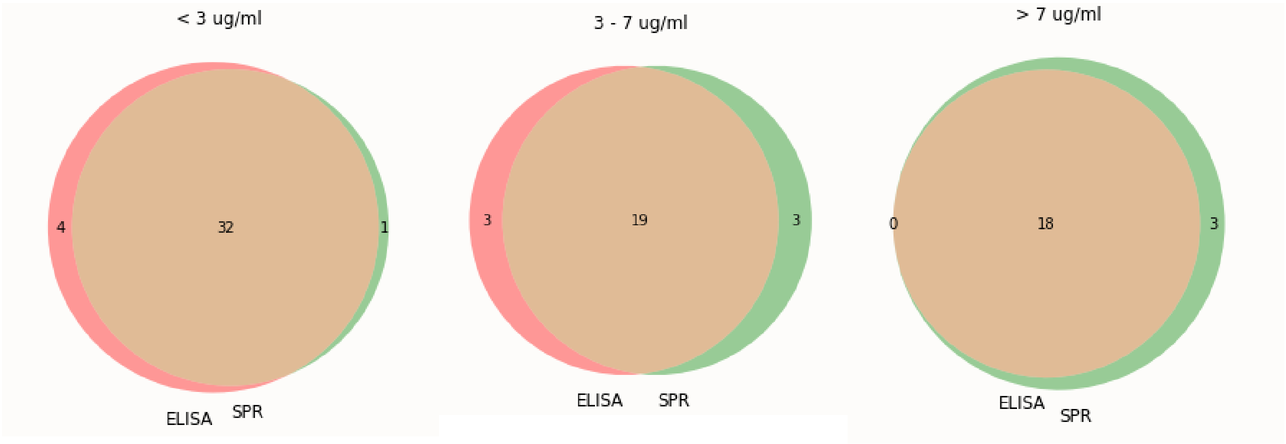

Suppl. Fig 2 Venn diagrams showing the numbers of patients detected by ELISA only (red), SPR only (green) or both ELISA and SPR (brown) for the different concentration ranges.
